# Exploring public attention and sentiment toward carbon neutrality: evidence from Chinese social media Sina Weibo

**DOI:** 10.3389/fpsyg.2023.1200824

**Published:** 2023-06-08

**Authors:** Bo Wang, Zixiao Jiang, Dawei Cheng, Ziao Wang

**Affiliations:** ^1^School of Humanities, Social Science & Law, Harbin Institute of Technology, Harbin, China; ^2^School of Water and Environment, Chang’an University, Xi’an, China; ^3^Key Laboratory of Subsurface Hydrology and Ecological Effects in Arid Region, Chang’an University, Xi’an, China; ^4^School of Energy Science and Engineering, Harbin Institute of Technology, Harbin, China

**Keywords:** carbon neutrality, public attention, public sentiment, social media, social psychology

## Abstract

**Introduction:**

Exploring the public’s cognition toward carbon neutrality is conducive to improving the quality and effectiveness of policymaking, and promoting the realization of carbon neutrality goals. This study aims to explore the public’s attention and sentiment toward carbon neutrality from the perspective of social psychology.

**Methods:**

Using posts on carbon neutrality from the Chinese social media platform Sina Weibo as the data source, this study uses statistical analysis, the Mann-Kendall method, keyword analysis, the BERT model, and the LDA model to explore public attention and sentiment.

**Results:**

The results show that: (1) men, people living east of the Hu line (economically developed regions), and the public in the energy finance market are more concerned about carbon neutrality; (2) high public attention and great dynamic changes in public attention toward carbon neutrality could be trigged by highly credible government or international governmental organizations’ information; (3) public sentiment toward carbon neutrality is mostly positive; however, specific topics affect public sentiment differently.

**Discussion:**

The research results contribute to policymakers’ better understanding of the trend of public attention and sentiment toward carbon neutrality, and support improvements in the quality and impact of policymaking.

## 1. Introduction

The impact of climate change on the ecosystem is an environmental issue facing the global community, with enormous implications for the economy, politics, and human society (Ide et al., 2020), with a consensus that all countries need sustainable development to address climate change (Schmidt-Traub et al., 2017; Xu et al., 2020; Cohen et al., 2021). Many countries signed the Paris Agreement to maintain global average temperature well below 2° Celsius (C), which is above pre-industrial levels (Lawrence et al., 2018; Sigmond et al., 2018). China has also committed to achieving peak carbon dioxide emissions by 2030 and carbon neutrality by 2060 (Li P. et al., 2022; Yang et al., 2022). In this background, the Chinese government has issued a variety of policies to achieve carbon emission reduction (Huang W. Q. et al., 2022; Xu et al., 2022).

When creating policies, policymakers do their best to explore the public’s cognition and take public perceptions into account to ensure smooth policy implementation (Li X. et al., 2022; Piselli et al., 2022). By considering the public attention and sentiment toward carbon neutrality, the quality and impact of policies that promote environmentally friendly behavior among citizens will improve (Li L. et al., 2021; Huang H. et al., 2022). For that reason, this study examines public attention and sentiment toward carbon neutrality, which, as mentioned, will help to adjust the appropriate carbon neutrality policies and improve the quality and impact of policy decisions. This analysis will contribute to the public cognition on carbon neutrality policies and help policymakers adjust their strategies to achieve carbon neutrality goals.

Current research on public attention and sentiment toward carbon neutrality focuses on several subfields. Huang H. et al. (2022) discuss the public’s attention to green consumption, demonstrate the importance of promoting Chinese residents’ low-carbon lifestyle, and suggest recommendations for the public to practice carbon-neutral behavior. Liu and Hu (2019) analyze public attention and sentiment toward green buildings, discuss the importance of energy conservation and consumption reduction in the building sector, and provide policy suggestions. Li L. et al. (2021) explore the status and trends of Chinese public attention and sentiment toward recycled water, and find that public perception to recycled water can be effected by government. Gong et al. (2022) demonstrate public attention, perception, and attitude toward nuclear energy, integrate the public’s regional attributes, social attributes, attitudes, and other aspects, and provide reference suggestions for government policies. However, in general, previous studies lack an overall analysis of public attention and sentiment toward carbon neutrality specifically, as well as a systematic explanation of the social psychological mechanism of public attention and sentiment toward carbon neutrality.

To fill this knowledge gap, this study aims to explore public attention and sentiment toward carbon neutrality from the perspective of social psychology, drawing on the Heuristic-System Model (HSM) and the Technology Acceptance Model (TAM) to address this research question. The HSM theory provides a theoretical explanation for individual information processing, dividing the factors that affect public psychology into heuristic cues and systemic cues (Chaiken, 1980; Chaiken and Maheswaran, 1994; Sarkar et al., 2023). Compared to HSM, TAM clearly defines the boundary between individual perception (a subset of public attention) and attitude (a subset of public sentiment) and proposes that individuals under stimulation by external variables will first develop perception, then attitude, and finally behavioral intention (Davis, 1989; Liu and Hu, 2019). By differentiating between heuristic cues (information source credibility) to trigger public attention and systematic cues (topics of the information content) to trigger public sentiment, HSM and TAM offer an appropriate theoretical framework to explore public attention and sentiment toward carbon neutrality.

The existing analysis of public attention and sentiment is primarily conducted through specialized interviews or surveys (Ho et al., 2018; Linzenich et al., 2020; Ho et al., 2022). Surveys and polls, however, must cover a wide range of target groups to obtain representative results, which is often time-consuming and costly. In addition, respondents may be biased due to the different environments of the survey and the design features of the questionnaire. With the continuous development of computer technology, more scholars are using social media platforms to conduct research (Lee and Chung, 2019; Zheng et al., 2019; Li L. et al., 2021), adopt semantic network analysis (Piselli et al., 2022), perform keyword analysis (Gong et al., 2022) and deep learning (Huang H. et al., 2022), as well as other methods to process data to obtain more objective and real results.

This study contributes to the existing literature in the following ways: (1) A new theoretical framework is proposed, drawing on the HSM and TAM theories to propose a social psychological model, clarify the internal mechanism of public attention and sentiment, and explore the theoretical boundary of HSM and TAM; and (2) this is one of the first studies to observe the public’s attention and attitude toward carbon neutrality from the perspective of social psychology. These research results can help policymakers understand public psychological tendencies and support improvements in the quality and impact of policymaking.

The structure of this paper is as follows: Section 2 proposes the process of public attention and sentiment formation based on HSM theory and TAM theory, and establishes research hypotheses based on heuristic cues (credibility of the information source) and systemic cues (topics of information content), according to the new theoretical framework. Section 3 presents the data and the research method, while Section 4 analyzes the results of the data processing on public attention and sentiment towards carbon neutrality, and Section 5 concludes the paper and makes corresponding policy suggestions.

## 2. Theoretical mechanisms and research hypotheses

### 2.1. Public attention and sentiment formation process based on the social psychological model

The HSM assumes that information processing is the preliminary stage of individual perception and attitude formation, and explains the process of individual information processing (Trumbo, 2002; Son et al., 2020). According to the HSM, people have two information processing modes: heuristic processing and systematic processing (Chaiken, 1980; Eagly and Chaiken, 1993; Sarkar et al., 2023). Heuristic processing means that a person makes quick judgments about heuristic clues of information based on experience and intuition without too much cognitive effort. Systematic processing means that a person evaluates information based on rationality and logic, which requires many cognitive resources. Factors influencing information processing include heuristic (non-content) and systematic (content) cues (Chaiken, 1980; Zhang et al., 2014; Sarkar et al., 2023). According to the study of Chaiken (1980) and Zhang and Watts (2008), heuristic (non-content) cues tend to correlate highly with intuition and experience, leading to heuristic behaviors, while systematic (content) cues are associated with rational judgment leading to systematic behaviors. The credibility of the information source is typical non-informative content cues that trigger heuristic processing. The topics of information content are systematic (content) cues that initiate systematic processing. Chaiken and Maheswaran (1994) consider that the information processing results are shown as heuristic dominance or systematic dominance. Therefore, the heuristic cues that trigger the onset of heuristic processing can be considered dominant heuristic variables. The systematic cues that cause the onset of systematic information processing can be considered dominant systematic variables.

Compared to information processing in social psychology, which is mainly covered by the HSM, the TAM assumes that perception influenced by external variables determines the formation of individual attitudes and then the intention to use (Davis, 1989; Chen et al., 2017; Li L. et al., 2021). The process of perceiving external variables is essentially the development of information processing. According to Chaiken and Trope (1999), a person is a “cognitive miser” when they usually make behavioral decisions with minimal cognitive effort. In the process of information processing, first heuristic processing is initiated, based on experience and intuition, and systematic processing is then initiated, based on rationality and logic. Depending on the differences in information cues, individuals show heuristic dominance or systematic dominance. When heuristic processing is dominant, intuition and experience help individuals perceive information. When systematic processing prevails, the individual logically and rationally analyzes the perceived information and forms an attitude, which is divided into positive and negative (Huijts et al., 2012; Fergen and Jacquet, 2016; Shi et al., 2023).

**Figure 1:**
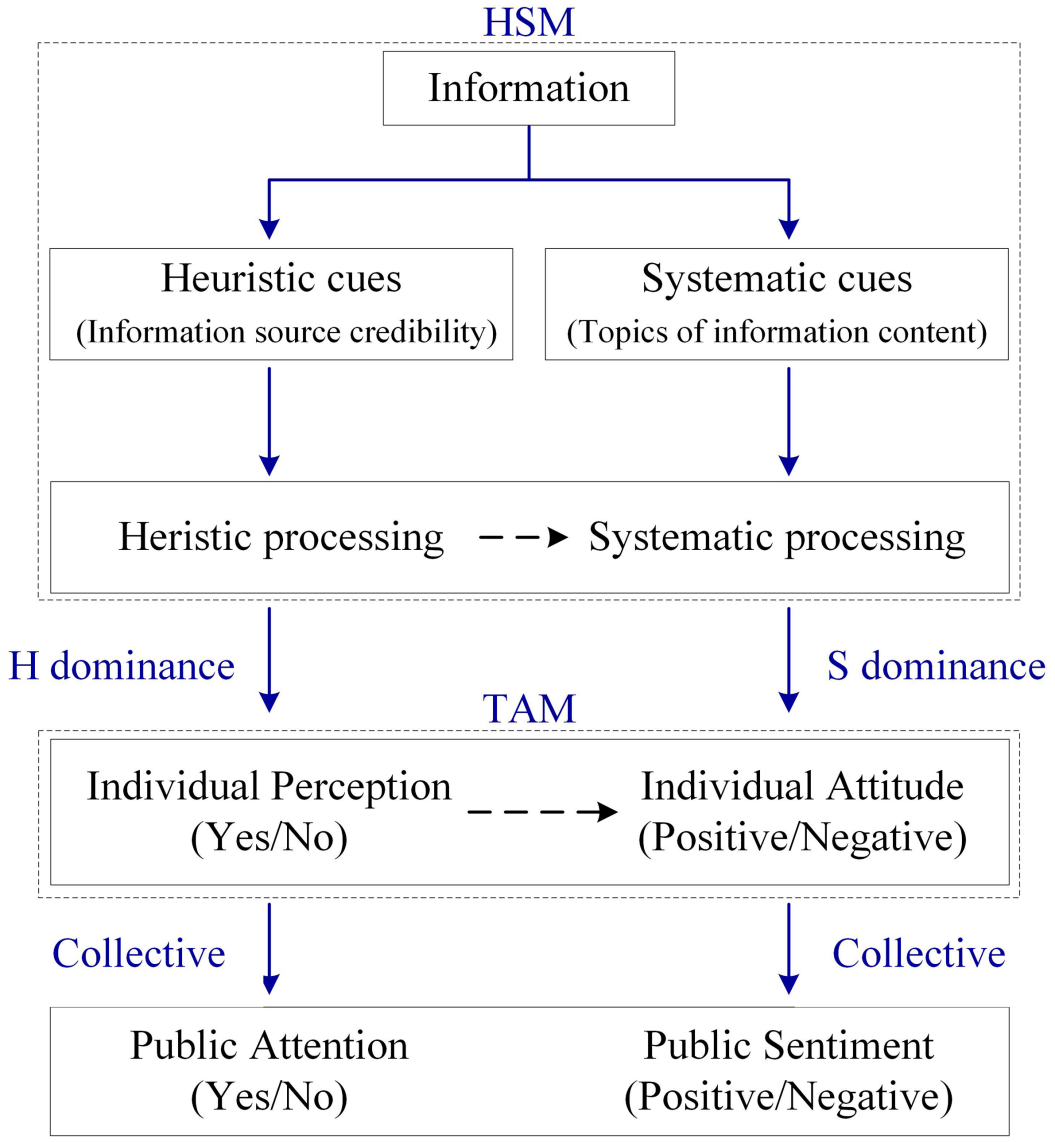
Public attention and sentiment formation process.

Public attention is the collective attention of individuals to a “common focus” (Adut, 2012). In collective attention, the public develops an opinion on social issues, divided into positive or negative (Park, 2019; Gong et al., 2022). In other words, public attention is a collection of individual perceptions of the same common focus. Public sentiment is a collection of individual attitudes toward the same common focus. Therefore, heuristic cues that trigger heuristic processing trigger public attention, and heuristic cues can be considered public attention variables. Systematic cues that trigger systematic processing trigger public sentiment, and systematic cues can be considered public sentiment variables. The process of formation of public attention and public sentiment is shown in Figure 1.

**Figure 2:**
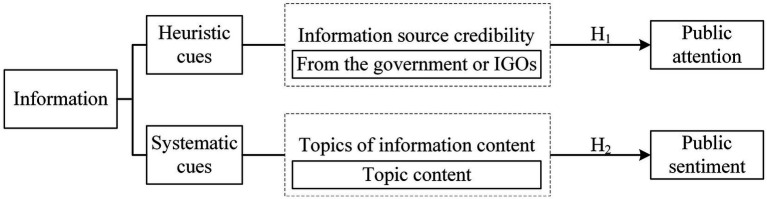
Research framework.

In this study, heuristic cues are non-content cues, including the credibility of the information source (from the government or international governmental organizations), which mainly trigger public attention toward carbon neutrality. Systematic cues are content cues, defined in this study as the topics of information content (topic content), which mainly trigger public sentiment toward carbon neutrality. Based on this, this study proposes a new research framework, as shown in Figure 2.

### 2.2. Public attention and sentiment toward carbon neutrality

#### 2.2.1. Heuristic cues

Depending on the different characteristics of social organizations, information sources can be divided into four categories: individuals, non-governmental organizations, media, governments, and international governmental organizations (IGOs) (Ho et al., 2018; Gong et al., 2022). Information source credibility is an important part of the information sources (Zhang et al., 2014; Shrivastava et al., 2021), and is an important factor in attracting public attention as heuristic cues. Compared to other sources of information, governments, and IGOs are the more credible sources of information and are considered objective sources (Ho et al., 2018; James and Petersen, 2018). Currently, China’s environmental policy depends on the government, and the carbon neutrality system is top-down (Wu et al., 2022). In addition, carbon neutrality was highlighted in the debate of the 75th Session of the United Nations General Assembly (organized by IGOs) (Yang et al., 2022). Thus, government and IGO information source credibility is higher (Ho et al., 2018; James and Petersen, 2018), and will raise Chinese citizens’ attention toward carbon neutrality, as well as have a greater effect on the dynamic of public attention. Therefore, this study proposes the first hypothesis.

*Hypothesis 1 (H1)*: More public attention and dynamic changes of public attention toward carbon neutrality could be trigged by highly credible government or IGOs information.

#### 2.2.2. Systematic cues

According to the research model proposed in Section 2.1, information content are variables that form public sentiment. When people expend sufficient logic and rationality to process information content related to carbon neutrality, systematic processing is initiated. Systematic processing leads individuals to evaluate the information content related to carbon neutrality and form different issue categories for carbon neutrality. In these evaluations, positive and negative public sensations are formed (Liu and Hu, 2019; Gong et al., 2022; Huang H. et al., 2022). If the public accepts carbon neutrality, they will develop positive feelings. Conversely, negative attitudes will result. Content about carbon neutrality covers many topics, such as finance, politics, technology, and society, and the sentiment of the text content varies according to the category of the topic. Therefore, this study proposes the second hypothesis.

*Hypothesis 2 (H2)*: Different topics pertaining to carbon neutrality have different effects on public sentiment.

## 3. Data and research method

**Figure 3:**
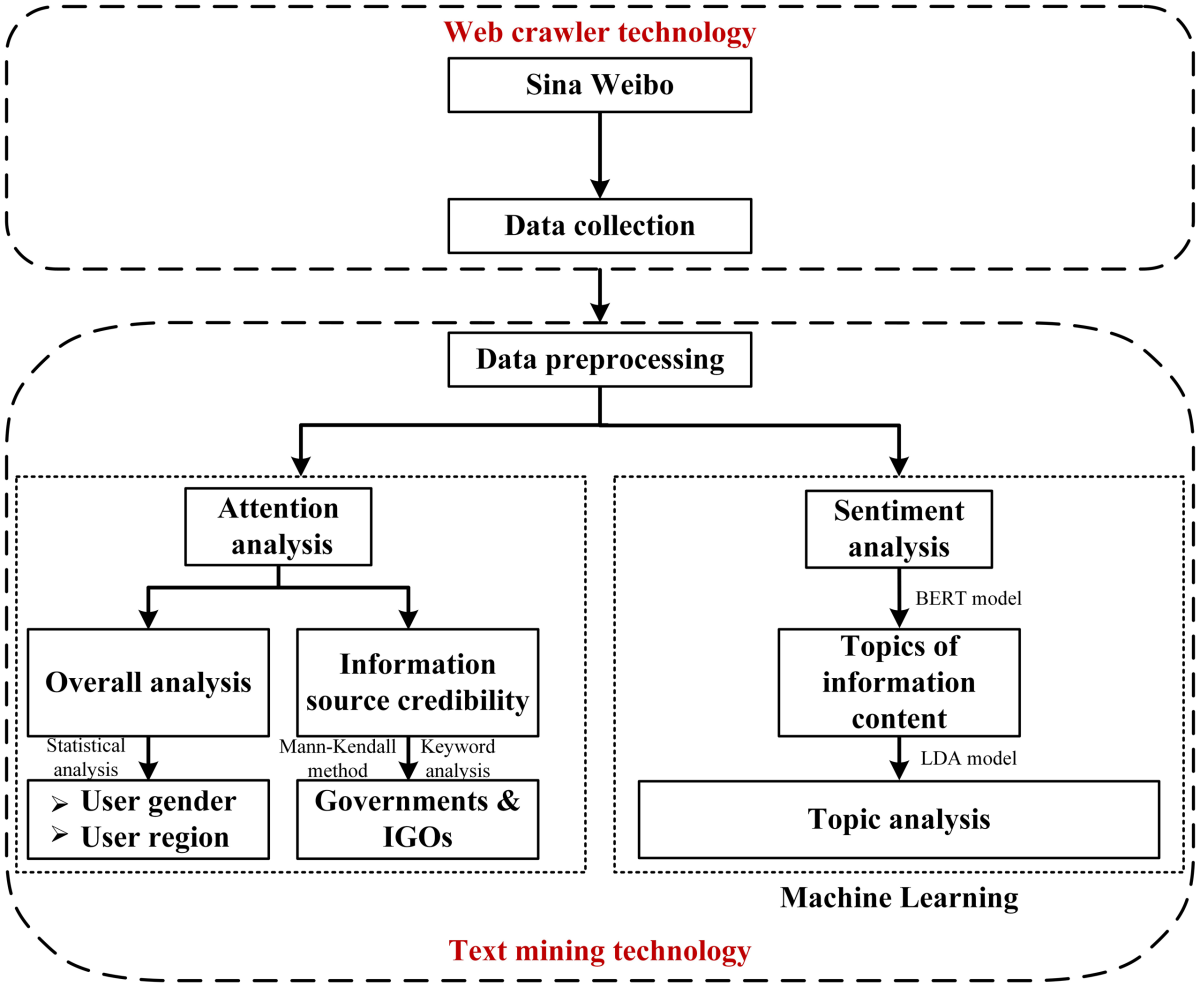
Research methodology.

In this study, web crawler technology was used to obtain original data from Sina Weibo, a Chinese microblogging website, and text mining technology was used to process the unstructured data. In this process, the public attention and sentiment toward carbon neutrality were investigated based on the formation process proposed in Section 2. The statistical analysis, the Mann-Kendall method, and keyword analysis were used to investigate the public’s attention toward carbon neutrality from the perspective of information source credibility. Then, the Bidirectional Encoder Representation from Transformers (BERT) model and the Latent Dirichlet Allocation (LDA) topic model were used for sentiment analysis and topic analysis, respectively, to examine public opinions toward carbon neutrality from the perspective of information content. The concrete research methodology is shown in Figure 3.

### 3.1. Data source and collection

As one of the most popular social networking platforms in China, Weibo had 224 million daily active users in 2020 (Weibo Official, 2021). Compared to traditional methods of data collection, such as questionnaires and interviews, the analysis of data from the social media platform Sina Weibo is more objective and scientific. This study used posts containing “carbon neutrality” on Sina Weibo as the data source to objectively and scientifically analyze Chinese public attention and sentiment toward carbon neutrality; it adopted web crawler technology in python version 3.9.7 to collect data.

The data acquisition process was as follows: (1) Simulation user login: we logged in to Weibo with our account to obtain the webpage source code and wrote python programs to simulate user login; (2) Keyword search: this research conducted an advanced search for the keyword “carbon neutrality” to obtain the initial link from January 1, 2020, to August 31, 2022; (3) Content collection: we collected information on carbon neutrality posts, including username, published content, publishing time, number of retweets, number of comments, and number of likes. At the same time, we collected information about the users who published relevant posts, including the description of the user, gender, region, date of birth, number of followers, and number of followers; and (4) Data storage and export: after collecting all contents, data was stored and exported to an Excel file.

### 3.2. Text mining method

In this study, natural language processing (NLP) techniques were mainly used to analyze the text data obtained from Sina Weibo, with the individual steps described below.

#### 3.2.1. Data pre-processing

The text data were pre-processed to prepare them for the following NLP task. First, we removed disordered, missing, and duplicate data. Texts with the same user ID, tweet content, and publication date were treated as duplicate posts and deleted, with only the first text information kept. Next, we deleted any meaningless content in the text, including URL links, special characters, usernames, and emoticons in the posts. Finally, 84,142 tweets were selected for public attention and sentiment analysis. Then, we manually annotated part of the dataset for sentiment analysis, using the label “0” for negative emotion and “1” for positive emotion. Finally, this study applied word segmentation. We merged the stop word lists from Harbin University of Technology, Baidu, and the Machine Intelligence Laboratory of Sichuan University to delete stop words in the texts and updated our stop word list based on the word segmentation results until obtained results met the requirements of the LDA model.

#### 3.2.2. Public attention analysis methods

##### 3.2.2.1. Statistical analysis of gender and region

To observe public attention toward carbon neutrality, this study quantified public attention by using the number of Weibo posts about “carbon neutrality.” that is, numerically, the public attention value is equal to the number of Weibo posts about “carbon neutrality.” Next, a quantitative analysis of non-content cues, including gender and region, was conducted. In analyzing the impact of gender differences on public attention to carbon neutrality, the ratio of attention given by different genders was used as a quantitative indicator to assess gender differences in public attention to carbon neutrality. It is expressed as the ratio of the different gender’s attention value to the total public attention value, as shown in Eq. (1):

(1) $$R_{i}=\frac{N_{i}}{N_{t}}\;$$

where $\;R_{i}\;$ is the ratio of i gender public attention; $\;N_{i}\;$ is i gender’s attention value, equals to the number of Weibo posts about “carbon neutrality” posted by gender i ; $\;N_{t}\;$ is total public attention value, equal to the sum of male and female posts.

In analyzing the impact of regional differences on public attention to carbon neutrality, the attention value of different regions was used as a quantitative indicator to assess regional differences in public attention to carbon neutrality. Here, the attention value of a region corresponds to the number of Weibo posts on the topic of carbon neutrality in that region. In this study, the attention value of different regions was transferred to the Chinese map to determine the different distribution of regional public attention for carbon neutrality.

##### 3.2.2.2. Mann-Kendall method

The Mann-Kendall (MK) mutation test is widely used to analyze the mutation of time series (Li J. et al., 2021). The MK mutation test is based on time series $\;\left\{x_{i}\right\}$ to construct order series $S_{k}$ defined in Eqs (2, 3).

(2) $$\begin{array}{c} S_{k}=\overset{k}{\underset{i=1}{\sum }}r_{i}\;\left(k=2,3,\ldots ,N\right) \end{array}$$

and there is,

(3) $$\begin{array}{c} r_{i}=\{\begin{array}{c} \;1,x_{i}>x_{j}\\ \;0,x_{i}<x_{j} \end{array}\;\left(j=1,2,\ldots ,i\right) \end{array}$$

where, $S_{k}$ is the accumulative number of $x_{i}>x_{j}$ . Under the assumption that the time series is random and independent, defining test statistic $UF_{k}$ in Eq. (4).

(4) $$\begin{array}{c} UF_{k}=\frac{S_{k}-E\left(S_{k}\right)}{\sqrt{Var\left(S_{k}\right)}}\;\left(k=1,2,\ldots ,N\right) \end{array}$$

where, $E\left(S_{k}\right)$ and $Var\left(S_{k}\right)$ are the mean and variance of $S_{k}$ respectively. When $x_{1},x_{2},\ldots x_{i}$ are mutually exclusive with the same continuous distribution, $E\left(S_{k}\right)$ and $V\left(S_{k}\right)$ is shown in Eqs (5, 6).

(5) $$\begin{array}{c} E\left(S_{k}\right)=\frac{N\left(N+1\right)}{4} \end{array}$$

(6) $$\begin{array}{c} V\left(S_{k}\right)=\frac{N\left(N-1\right)\left(2N+5\right)}{72} \end{array}$$

$UF_{k}$ is the standard normal distribution, and it is the statistical series calculated through the time series $\left\{x_{i}\right\}$ . When the absolute value of $\;UF_{k}$ is greater than or equal to 2.58, it passes the significance test with 99% confidence. If $\left|UF_{k}\right|>U_{\alpha }$ , indicates the series has an obvious trend. In addition, the time series $\left\{x_{i}\right\}$ are arranged in reverse order, and the above method is applied to the reverse order. Last, repeat the above process and multiply the calculated value by-1 to obtain $\;UB_{k}$ . If the intersection point between $UF_{k}$ and $\;UB_{k}\;$ curves is within the confidence interval, this point is the mutation point, and the corresponding time is the starting moment of mutation.

##### 3.2.2.3. Keyword analysis

After the MK mutation test, a keyword analysis for mutation points and top-ranking data is performed to investigate the specific reasons for the mutation of public attention. A word cloud is a visualization tool for text data that can effectively display high-frequency words in a text and help people quickly grasp key information in big data (Abdar et al., 2020; Piselli et al., 2022). In general, the size of the words in the word cloud is positively correlated with the frequency of their occurrence in the text, so it can intuitively show the meaning of some words. Keyword analysis is used to discuss the word frequency before and after the mutation and the 5 weeks with the most public attention in the time series to find important topics that can trigger a public discussion about carbon neutrality.

#### 3.2.3. Public sentiment analysis methods

##### 3.2.3.1. BERT model

Traditional sentiment analysis methods generally rely on sentiment dictionaries and statistical machine learning, which not only require a great deal of manpower, time, and energy to label a large amount of textual data, but also ignore bidirectional understanding of contextual representations, resulting in inaccurate sentiment classification of a text corpus. To solve these problems, a deep learning model known as BERT was used in this study. The Bidirectional Encoder Representation from Transformer (BERT) model is a language representation model proposed by Google AI Language in 2018, which is an important breakthrough in the field of NLP (Devlin et al., 2018). In this study, the Chinese BERT-wwm was used as a pre-training model, and then the BERT model was fine-tuned. Deep neural networks were used to extract the features of the language, and the algorithm is constantly optimized. In the training process, the highest accuracy reached 94.6%, and the best result was selected to classify the public sentiment on carbon neutrality.

##### 3.2.3.2. LDA model

To explore the reasons for the public’s different emotions toward carbon neutrality, we used the LDA model to conduct a thematic analysis of positive and negative sentiments, respectively. The LDA model was originally proposed by Blei, and is an unsupervised machine-learning technique for extracting latent topic information from text datasets. It is widely used in natural language processing due to its excellent performance in topic modeling (Gong et al., 2022; Huang H. et al., 2022). In this study, we extracted hidden topics, and the Python package “pyLDAvis” was applied to visualize the results of topic extraction. To determine the optimal number of topics, perplexity and visualization results of “pyLDAvis” were considered in this study. The lower the perplexity, the better the clustering effect, and the specific equation is given in Eq. (7).

(7) $$\begin{array}{c} Perplexity\left(D\right)=\exp\left[-\frac{\sum_{m=1}^{M}\log_{D}p\left(w_{m}\right)}{\sum_{m=1}^{M}N_{m}}\right] \end{array}$$

where D is the test set in the corpus; M is the number of documents; $N_{m}\;$ is the number of words in document M ; $p\left(w_{m}\right)$ is the probability of each word in the test set.

According to the principle of “pyLDAvis,” different bubbles represent different topics. The smaller the overlap of the bubbles, the better the effect of clustering. In this study, the optimal number of topics was determined by combining the results of the perplexity calculation and visual observation. Finally, we selected 10 high-frequency words as representatives of each topic and determined the topic name according to the semantic relationship between feature words.

## 4. Results and discussion

### 4.1. Public attention analysis

#### 4.1.1. Overall analysis of gender and region

**Figure 4:**
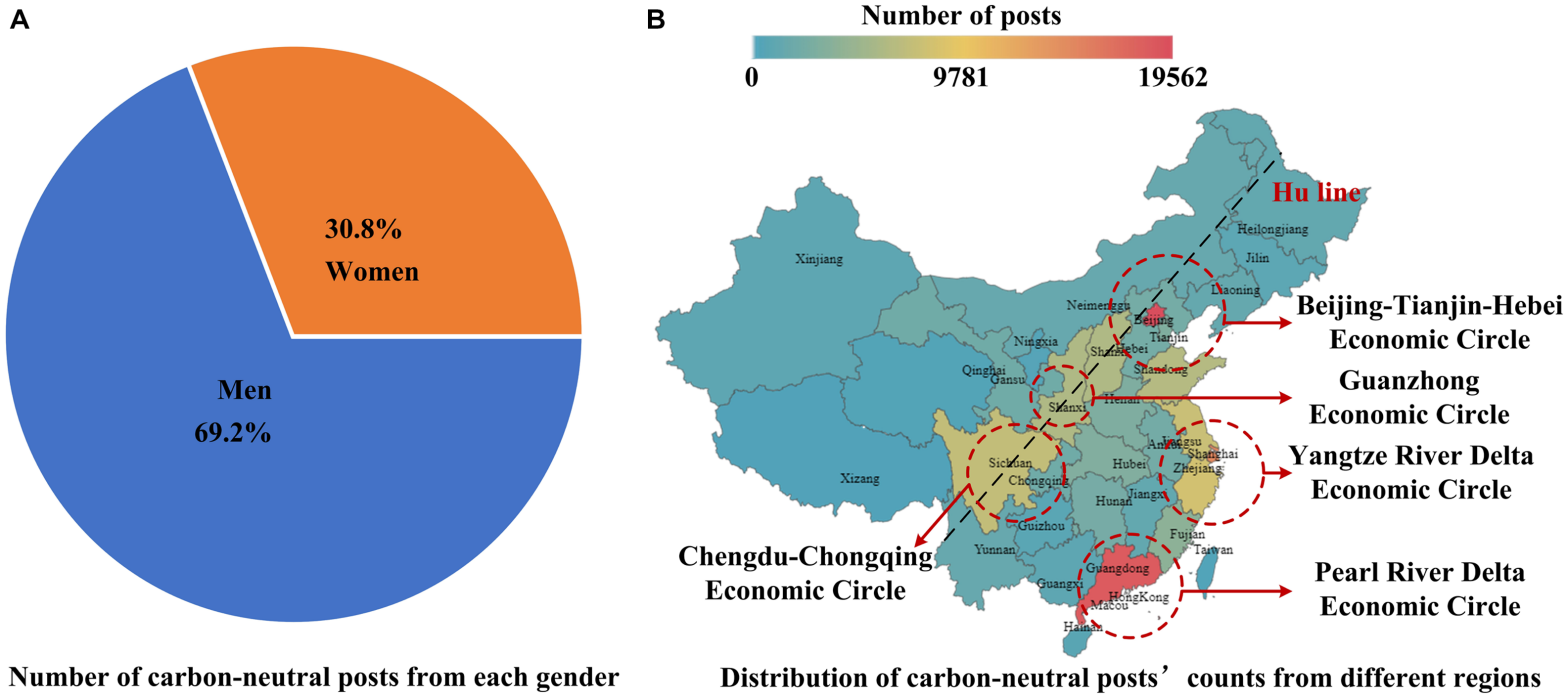
Overall analysis of gender and region. **(A)** represents the number of carbon-neutral posts from each gender. **(B)** represents the distribution of carbon-neutral posts’ counts from different regions.

Figure 4A shows the number of carbon-neutral posts from each gender, with the number of posts from men at 58,231 posts, which means that the attention value of men is 58,231. The number of posts from women was 25,911, which means that the attention value of women is 25,911. The results show that 69.2% of men pay attention to carbon neutrality. Some existing studies believe that women are more concerned about environmental issues than men (May et al., 2021), while others believe the opposite is true (Xiao and Hong, 2010). This study provides evidence that Chinese men are indeed more concerned about carbon neutrality than Chinese women. This may be because Chinese men have a higher level of environmental knowledge because of their higher education level (Hong and Xiao, 2007; Xiao and Hong, 2010).

Figure 4B shows the distribution of carbon-neutral posts from different regions, which is the regional distribution of the public attention value for carbon neutrality. From this perspective, different locations of information recipients lead to different levels of public attention to carbon neutrality. The Hu line is the geographical dividing line of uneven regional economic development in China. On the eastern side, more than 94% of the population lives on less than 44% of the land area, while on the western side, less than 6% lives on more than 56% of the land area (Chen et al., 2016). According to the Hu line, the areas with greater public attention are mainly located east of the Hu line, which is consistent with the geographical division of China’s economy. There is a large population east of the Hu line and a high degree of economic development where people pay more attention to carbon neutrality, while the west is the opposite.

From the perspective of urban clusters, the regions where the public paid special attention to carbon neutrality are mainly concentrated in the Beijing-Tianjin-Hebei Economic Circle, the Yangtze River Delta Economic Circle, the Pearl River Delta Economic Circle, the Chengdu-Chongqing Economic Circle, and the Guanzhong Economic Circle. These five economic circles undertake the important task of guiding China’s economic development and fulfilling the carbon reduction goals earlier than other regions, thus, paying more attention to the issue of carbon neutrality.

The three regions with the greatest public attention toward carbon neutrality are Beijing, Guangdong Province, and Shanghai, which belong to the core cities and provinces of the Beijing-Tianjin-Hebei Economic Circle, the Yangtze River Delta Economic Circle, and the Pearl River Delta Economic Circle, respectively. Users in Beijing posted 39,124 posts, and were the region with the greatest public interest in carbon neutrality. As the political center of China, the public in Beijing is more sensitive to carbon-neutral policies. Users in Guangdong wrote 18,478 posts, ranking second. Guangdong is one of the first pilot provinces for low-carbon transformation and one of the seven pilot provinces for carbon emissions trading. Shanghai ranked third, with 14,854 posts over 2 years. As one of the important leaders of green finance in China, Shanghai has a considerable scale of green investment.

These findings are consistent with previous studies about the public’s concern for environmental issues (Liu and Hu, 2019; Huang H. et al., 2022). In the studies by Liu and Hu (2019), Huang H. et al. (2022), and Huang W. Q. et al. (2022), people in the eastern region focused more on environmental issues than people in the western region, and people from economically developed regions were more concerned with energy transition. It is probably because local governments often implement carbon-neutral policies earlier and take measures to regulate the public’s carbon-neutral behavior in economically developed regions (Huang H. et al., 2022).

According to the overall analysis, the results show that men and people from economically developed regions pay more attention to carbon neutrality. Thus, policymakers should raise awareness of carbon neutrality among women and people in economically underdeveloped regions.

#### 4.1.2. Public attention toward carbon neutrality from the perspective of information source credibility

**Figure 5:**
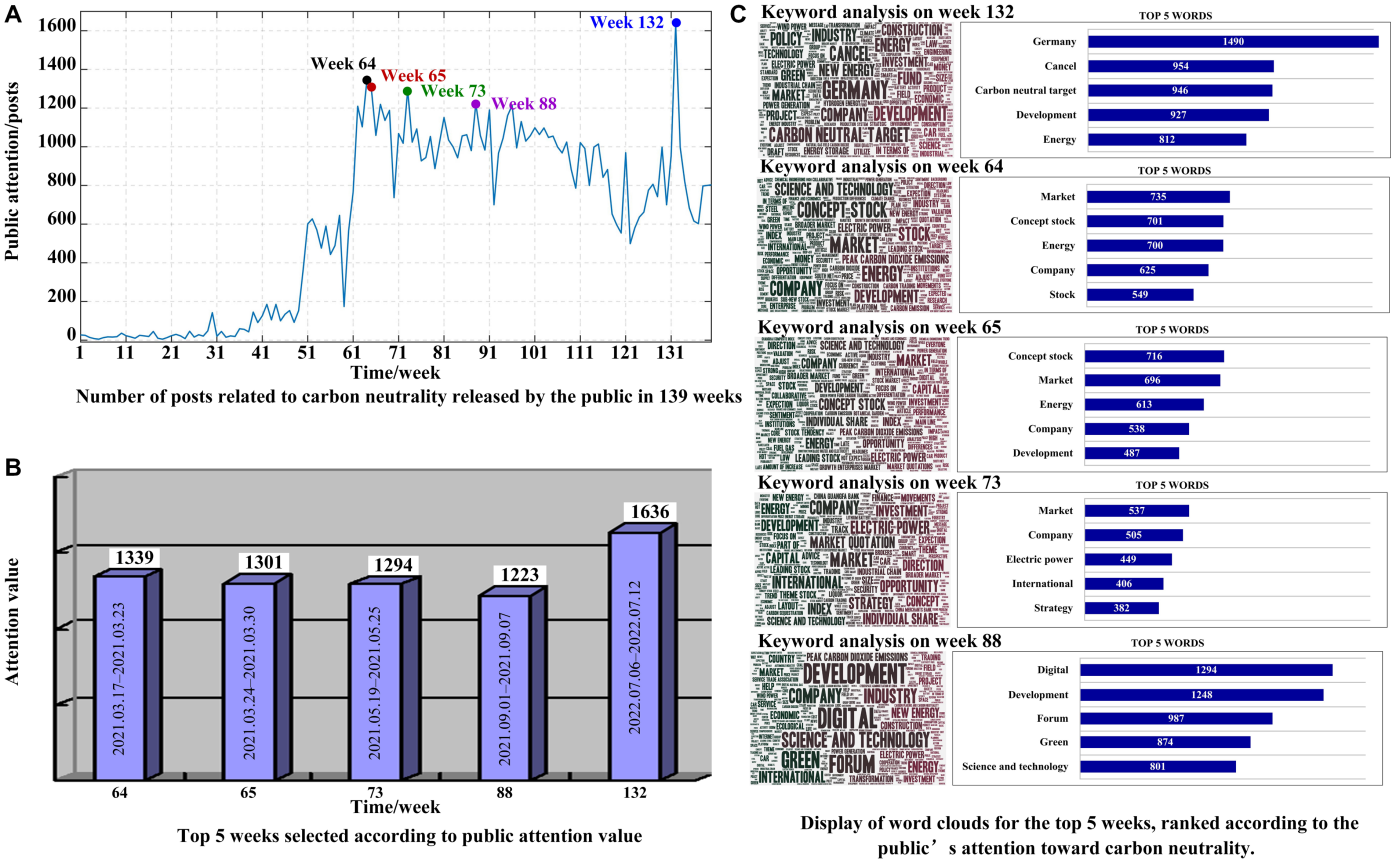
The dynamics of public attention to carbon neutrality and the reasons for high public attention. **(A)** represents the number of posts related to carbon neutrality released by the public in 139 weeks. **(B)** represents the top 5 weeks selected according to public attention value. **(C)** represents word clouds for the top 5 weeks, ranked according to the public’s attention toward carbon neutrality.

We next examined the dynamics of public attention to carbon neutrality and analyzed the reasons for high public attention and dynamic change. As shown in Figure 5A, the horizontal axis is the time (unit week), the vertical axis is the number of posts published by users (public attention value), and the blue line is the public attention value of users every week from January 1, 2020, to August 31, 2022.

To explore the reasons for the high level of public attention to carbon neutrality, this study selected the top 5 weeks ranked by value of public attention. According to the Figure 5B, public attention toward carbon neutrality in week 132 was the highest, and the value of public attention was 1,636. The following rankings are week 64, week 65, week 73, and week 88, and the value of public attention is 1,339, 1,301, 1,294, and 1,223, respectively.

To investigate the keywords discussed in these weeks, word cloud analysis was performed, which shows the keywords of public discussion, as shown in Figure 5C. According to Figure 5C, the reason that the Chinese public’s attention to carbon neutrality is much higher in week 132 was due to the discussion surrounding the cancelation of Germany’s 2035 carbon neutrality target. However, the media and the public misunderstood the information published on the official website of the German Bundestag and that cancellation was not to occur. Nevertheless, the news in China attracted public attention to the issue of carbon neutrality and became a trending topic on Weibo on July 11, 2022 (Finance Net Official, 2022). This phenomenon shows that a government announcement will lead to significant changes in public attention toward carbon neutrality.

The high-frequency words in week 88 are “digital,” “development,” “forum,” “green,” “science,” and “technology.” Examining the events in week 88, it can be seen that the 1st Chinese Digital Carbon Neutrality Summit Forum was held, hosted by the Cyberspace Administration of China, the National Development and Reform Commission, the Ministry of Ecology and Environment, the People’s Government of Sichuan Province, and other national government agencies (International Business Daily Official, 2021). With the theme of “Digital Power, Green Development,” the forum generated attention toward carbon neutrality. Therefore, carbon-neutral messages published or disseminated by governments can draw public attention to carbon neutrality.

It can be seen that the five most frequent words appearing in Week 64, Week 65 and Week 73 include “market,” “concept stock,” “energy,” “company,” and “stock” in the second rank consecutively; “concept stock,” “market,” “energy,” “company” and “development” in the third rank consecutively; “market,” “company,” “electric power,” “international,” and “strategy” in the fourth rank consecutively. The results show that the keywords from the second week to the fourth week are very similar and mainly include “market,” “energy,” “concept stock,” and so on, which are closely related to the energy financial market. This result reveals that the public pays close attention to carbon neutrality in the energy finance market. Unlike in previous studies, however, this is an exploratory finding. Therefore, policymakers need to consider the movement of energy financial markets and understand the public’s psychosocial cognition when formulating relevant policies.

**Figure 6:**
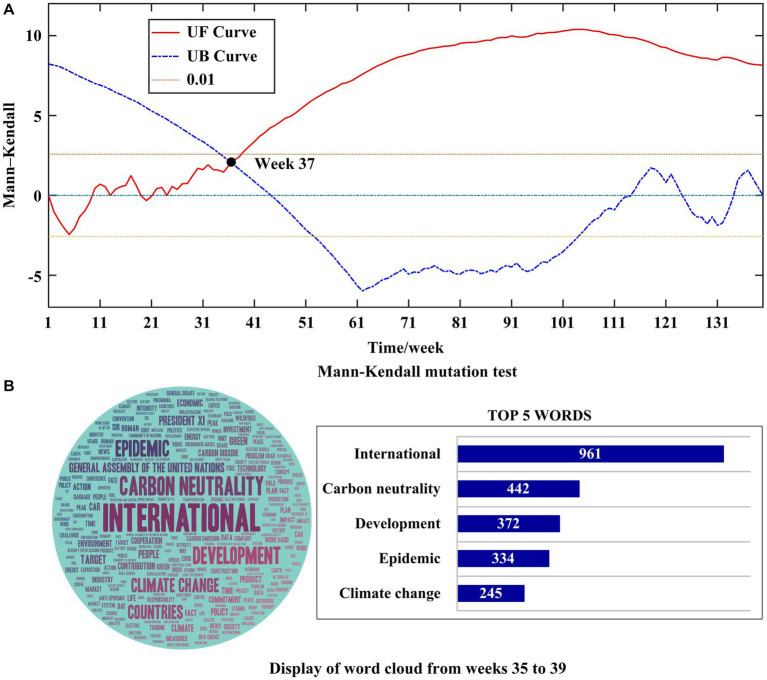
The reason for great dynamic changes of public attention toward carbon neutrality. **(A)** represents the result of Mann-Kendall mutation test. **(B)** represents word cloud from weeks 35 to 39.

Next, the MK mutation test was used to analyze the mutation of public attention toward carbon neutrality, as shown in Figure 6A, which shows the results of $UF_{k}$ (red line) and $UB_{k}$ (blue line) curves. The intersection point between $UF_{k}$ and $UB_{k}$ curves is within the confidence level interval [−2.58 2.58]. The intersection point is in week 37, suggesting that public attention to carbon neutrality may suddenly increase from week 37. To clarify the reasons for the mutation as much as possible, we focused on the keyword analysis of all posts from week 35 to 39 (August 26, 2020–September 29, 2020), that is, 2 weeks before and 2 weeks after the mutation.

In Figure 6B, the left is the word cloud map, and the right is the bar chart of the top five-word frequency rankings. As you can see, the word frequency of the word “international” is significantly higher than in other words, suggesting that the proposal of the international carbon neutrality target was the reason for the sudden increase in public attention to carbon neutrality. The general debate of the 75th Session of the United Nations General Assembly was been held in New York City on September 22, 2022. At the debate, China committed that they would achieve peak carbon dioxide emissions by 2030 and carbon neutrality by 2060. President Xi Jinping also proposed to accelerate the formation of green development models and lifestyles, pay attention to climate change, and engage in global green and low-carbon transformation (Yang et al., 2022). Therefore, the international carbon neutrality goal proposed in the IGOs led to a sudden surge of public interest in carbon neutrality in China.

Based on the analysis of the first and fifth rank of public attention and the mutation point, it can be concluded that information from highly credible governments or IGOs is the reason for higher public attention toward carbon neutrality and the great dynamic changes of public attention toward carbon neutrality. Therefore, H1 is verified. This is consistent with Ho et al. (2018), who suggest that governments and IGOs have access to exclusive information related to energy, and the public trusts messages delivered by governments and IGOs. Information from government and IGOs with high credibility are more effective at attracting public attention toward carbon neutrality. Therefore, as policymakers, it is necessary to use information cues from governments and IGOs to promote carbon neutrality policies.

### 4.2. Public sentiment analysis

**Figure 7:**
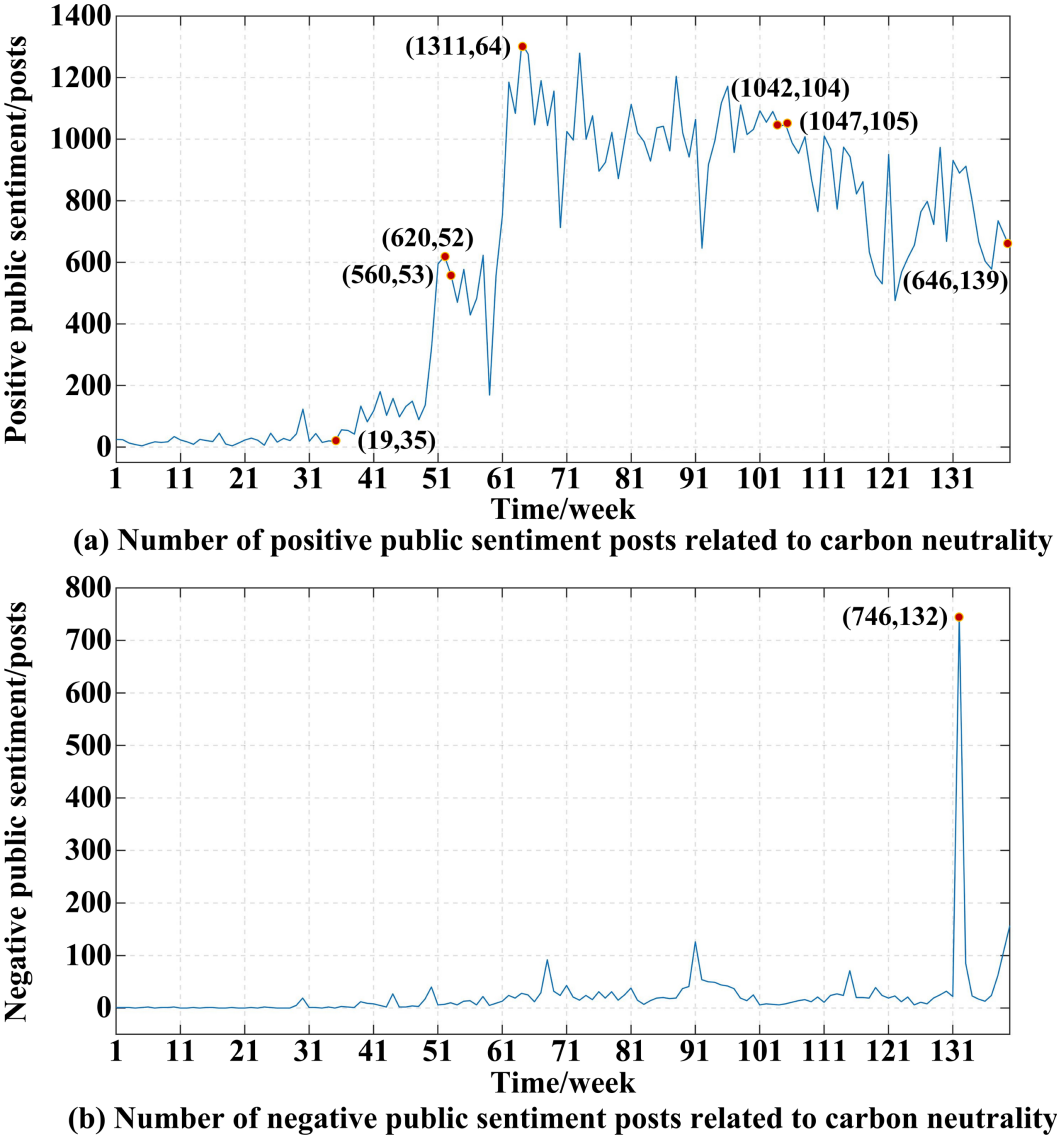
Number of positive and negative public sentiment posts related to carbon neutrality released by the public in 139 weeks. The first number in () denotes the number of posts, and the second number denotes the observed week. **(A)** represents the number of positive public sentiment posts related to carbon neutrality. **(B)** represents the number of negative public sentiment posts related to carbon neutrality.

To deeply explore the influence of different systematic cues on public sentiment in systematic processing, this study conducted sentiment analysis and topic analysis. In this study, the BERT model was used to classify 84,142 text data for sentiment classification. The results show that 80,913 posts have a positive sentiment towards carbon neutrality, which is 96.2%. It can be seen that the sentiment of the Chinese public toward carbon neutrality is positive. This finding is consistent with those of Gong et al. (2022) and Huang H. et al. (2022), who show that the public has a positive sentiment toward green consumption and clean energy. One explanation is that Chinese carbon neutrality system is top-down (Wu et al., 2022), and Chinese people trust Chinese government with high credibility and authority, so people have a positive sentiment toward carbon neutrality. Figure 7 shows the number of posts with positive and negative public sentiment per week from January 1, 2020, to August 31, 2022.

Figure 7A shows that the time trend of positive public sentiment toward carbon neutrality is consistent with the time trend of public sentiment toward carbon neutrality and that the overall trend is increasing. The total number of positive messages in public posts was lower in 2020 than in 2021, but public posts on carbon neutrality showed a significant upward trend from week 35 to 39 (August 26, 2020–September 29, 2020). This coincides with the week with the mutation weeks in Section 4.1.2. In September 2020, Chinese President Xi Jinping announced in the general debate of the 75th session of the United Nations General Assembly that China would peak carbon emissions by 2030 and be carbon neutral by 2060. This statement drew the attention of Chinese citizens to carbon neutrality and formed positive public sentiment. After that, the number of posts with a positive sentiment published per week increased from 19 in week 35 to 620 in week 52 (December 23, 2020–December 29, 2020), which was the peak of posts with a positive sentiment published in 2020. In 2020, Chinese citizens began to pay close attention to carbon neutrality, and positive public sentiment increased.

In 2021, positive public sentiment toward carbon neutrality rose, with weekly positive sentiment posts moving from 560 in week 53 (December 30, 2020–January 5, 2021) to 1,042 in week 104 (December 22, 2021–December 28, 2021). The week with the highest positive sentiment was week 64 (March 17, 2021–March 23, 2020) with 1,311 posts. Carbon neutrality and peak carbon dioxide emissions became focal points of public opinion in March 2021. After week 64, the number of carbon-neutral posts published each week in 2021 exceeded 600. Therefore, the concept of carbon neutrality was popular in 2021, with a high threshold value of positive public sentiment.

In 2022, there was widespread acceptance of carbon neutrality, but public sentiment toward carbon neutrality fluctuated. Compared to the 1,047 positive sentiment posts in week 105 (December 29, 2021–January 4, 2022), the number of positive sentiment posts in week 139 (August 30, 2022–August 31, 2022) was 646, and the total number of positive sentiment posts was slightly down. However, the number of positive sentiment posts published each week was higher than 450, indicating that people were still very interested in the subject of carbon neutrality.

Figure 7B shows that the number of negative public posts about carbon neutrality is low, less than 160, with the exception of week 132. The week with the most negative posts was week 132 (July 6, 2022–July 12, 2022) with 746 posts. This coincides with the week with the highest public attention in Section 4.1.2.

Next, we applied the LDA model to the positive and negative sentiment datasets to examine the specific topic content of Chinese citizens’ different attitudes toward carbon neutrality.

#### 4.2.1. Topic analysis in positive sentiment

**Figure 8:**
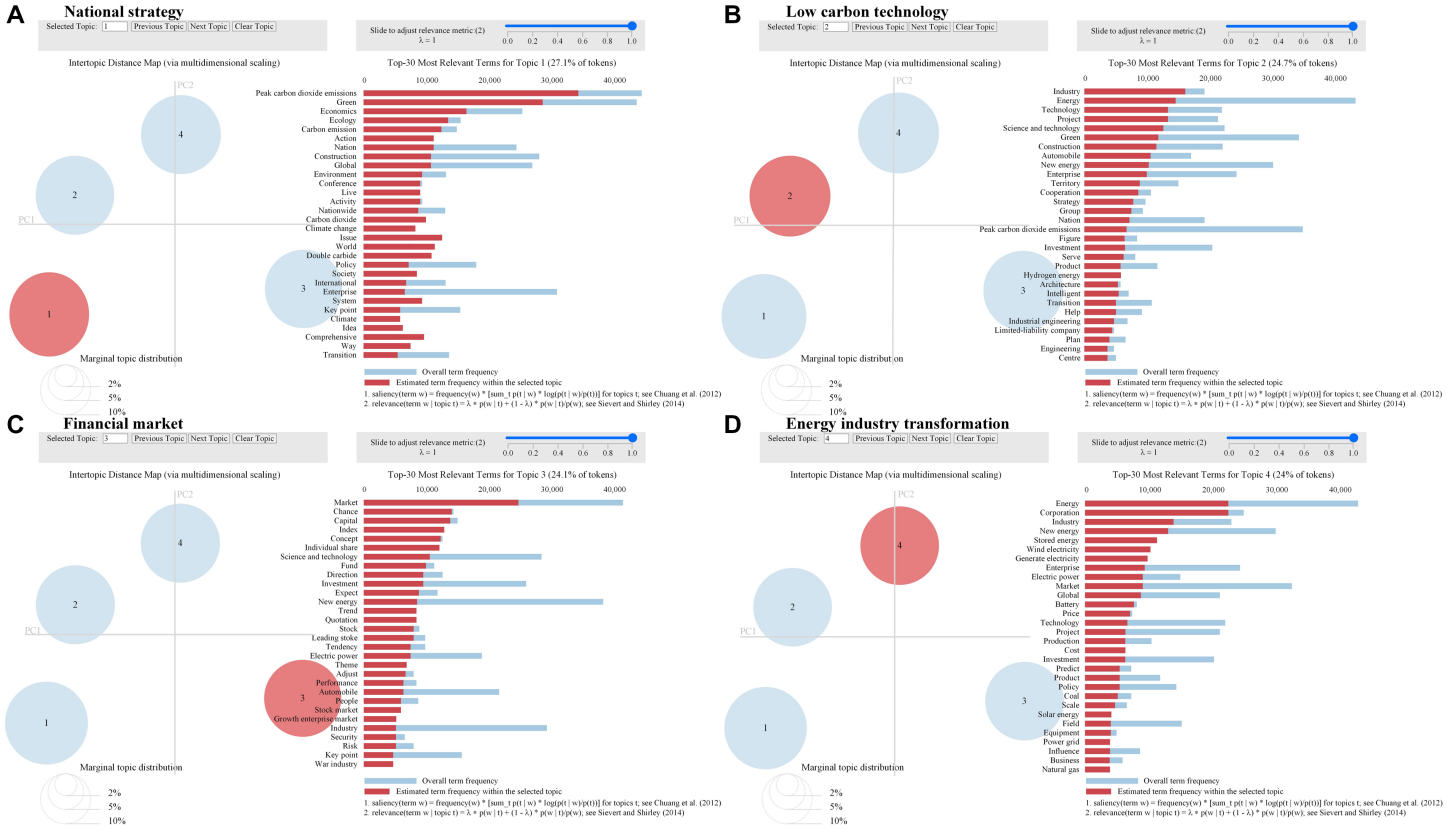
Topics on positive sentiment. **(A)** represents topic 1. **(B)** represents topic 2. **(C)** represents topic 3. **(D)** represents topic 4.

To analyze topic sentiment, the LDA model was first used to cluster the textual data of positive public sentiment. By calculating the perplexity and observing the visualization results, we set the optimal number of topics to 4. Next, the LDA model was used to train the text data with 50 iterations to obtain a topic-term matrix. Figure 8 shows different topics (bubbles on the left) and the top 30 feature words for each topic (bars on the right). In the bar chart, the light blue color represents the overall term frequency, and the dark red color represents the estimated term frequency within the selected topic. Finally, 10 high-frequency words are selected from the top 30 feature words as topic representatives. The topics of national strategy, low carbon technology, financial market, and energy industry transformation are determined based on the semantic relationship of the feature words. The results are shown in Table 1.

**Table 1:** Topics of positive public sentiment.

No.	Topic	Feature word
1	National strategy	Peak carbon dioxide emissions, green, economics, ecology, action, nation, construction, conference, policy, society, system
2	Low carbon technology	Industry, technology, science and technology, project, new energy, digital, product, hydrogen energy, intelligent, engineering
3	Financial market	Market, chance, capital, index, individual share, fund, investment, quotation, stock, performance
4	Energy industry transformation	Energy, corporation, stored energy, wind electricity, generate electricity, electric power, battery, solar energy, power grid, natural gas

Topic 1 indicates that China has promoted peak carbon dioxide emissions and carbon neutrality as a national strategy, which has aroused a positive public sentiment toward carbon neutrality. During the study period, China introduced a series of carbon neutrality policies, established carbon neutrality action plans, mentioned the dual carbon goal in many national meetings, and implemented carbon neutrality policies from economic, environmental, and other aspects. Carbon neutrality as a national strategic goal has increased public confidence in China’s mission to achieve carbon neutrality.

Topic 2 shows the words related to low-carbon technology, indicating that low-carbon technology innovation has triggered positive public sentiment toward carbon neutrality. During the period of this study, the Chinese paid more attention to the innovation of new energy, hydrogen, and other low-carbon technologies, and developed digital technologies and industrial intelligence to achieve the digitization of industry and low-carbon transformation. These low-carbon technological innovations have promoted changes in various industries, increased public confidence in the implementation of carbon neutrality goals, and formed positive public sentiment.

Topic 3 finds that words related to the financial market, which shows that the positive carbon-neutral financial market aroused positive public sentiment. Achieving carbon neutrality requires significant financial support, and encouraging and directing the participation of social capital is key to achieving a green transformation. During the period of this study, the financial market is paying close attention to carbon neutrality; the inflow of green and low-carbon capital is also increasing, and the financial market for carbon neutrality is constantly positive, leading to positive public sentiment in the financial market for carbon neutrality.

Topic 4 appears with words related to energy industry transformation, indicating that China’s energy industry transformation triggered positive public sentiment toward carbon neutrality. China’s energy industry is currently undergoing a revolutionary transformation; led by the strategic goal of carbon neutrality, it is moving in a green and low-carbon direction. Several energy companies are turning to renewable energy sources such as solar and wind power to replace traditional thermal energy. The transformation in the energy sector has boosted public confidence in achieving carbon neutrality.

#### 4.2.2. Topic analysis in negative sentiment

**Figure 9:**
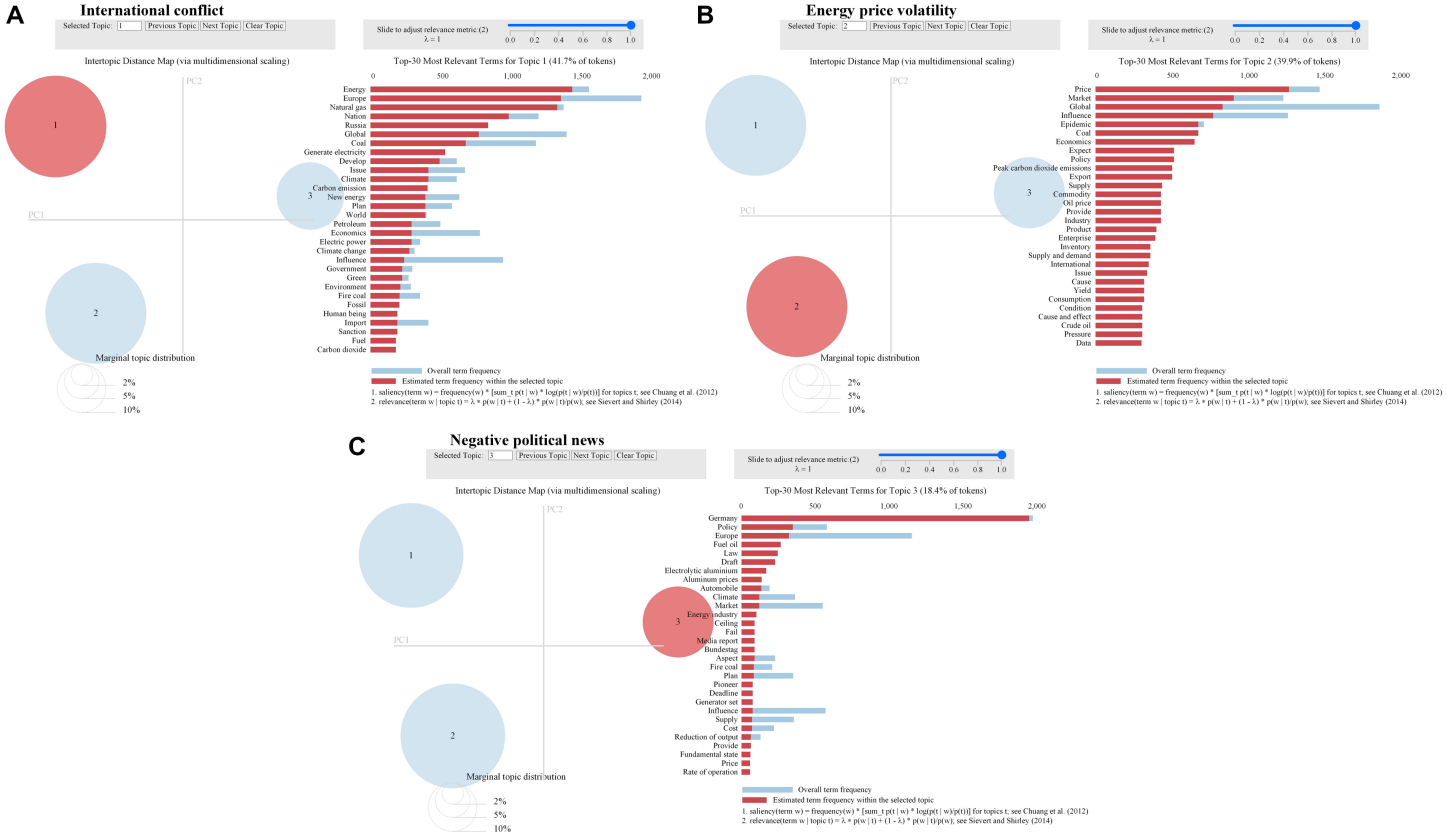
Topics on negative sentiment. **(A)** represents topic 1. **(B)** represents topic 2. **(C)** represents topic 3.

A similar method to the abovementioned one was adopted to analyze carbon-neutral negative sentiment text data. We determined the optimal number of topics as three to obtain the topic-term matrix, with Figure 9 showing the results of the visualization of pyLDAvis. Ten high-frequency words were selected from the top 30 feature words as topic representatives. The topics of international conflict, energy price volatility, and negative political news were determined based on the semantic relationship of the feature words. The results are shown in Table 2.

**Table 2:** Topics of negative public sentiment.

No.	Topic	Feature word
1	International conflict	Energy, Europe, natural gas, nation, Russia, problems, petroleum, influence, government, sanction
2	Energy price volatility	price, market, coal, export, supply, commodity, oil price, supply and demand, crude oil, pressure
3	Negative political news	Germany, policy, law, draft, climate, energy industry, media report, Bundestag, fire coal, plan

According to the results, topic 1 contained international conflict, reflecting the public’s worry about the energy crisis caused by the Russia-Ukraine conflict, which has disrupted a global response to climate change and energy transition (Carriquiry et al., 2022). The global energy game continues, leading to natural gas shortages in some areas. The international conflict is causing the Chinese public to lose confidence in carbon neutrality targets.

Topic 2 shows energy price volatility. China is highly dependent on foreign energy, and crude oil and coal are major energy imports in China. Changes in international energy prices have seriously affected China’s economic development, and the increase in energy prices caused by decarbonization has raised public concerns about “green inflation.” In this context, the energy supply is tight, and several regions in China have implemented power rationing measures. In 2021, the domestic “power outage wave” was triggered (Financial News Official, 2021), which resulted in public resistance to the carbon neutrality target.

Topic 3 includes negative political news with representative words. These discussions are related to erroneous media reports mentioned previously about Germany’s cancellation of the 2035 carbon neutrality target. It turns out that negative political news about energy shortages will lead to negative public sentiment toward carbon neutrality.

In general, different topics on carbon neutrality have different effects on public sentiment. This result supports H2 and is consistent with the results of the existing literature (Liu and Hu, 2019; Gong et al., 2022; Huang H. et al., 2022). However, the novel finding is that carbon neutral topics concerned with promoting the development of green energy, including national strategy, low carbon technology, financial market, and energy industry transformation, may generate positive sentiment among the public; however, carbon neutrality topics related to inadequate energy supply, including international conflict, energy price volatility, and negative political news, may evoke negative public sentiments. This finding is distinct from other studies in that we focus on specific topics related to public sentiment toward carbon neutrality and provide a summary of these topics at the end. Based on these results, policymakers need to introduce carbon neutrality policies to promote the development of green energy and focus on resolving the contradiction between energy supply and demand.

## 5. Conclusions and policy implications

This study focused on exploring public cognition toward carbon neutrality according to a new theoretical framework, which plays a critical role in the implementation of carbon neutrality policies and expanding of the theoretical boundaries of social psychological models. Policymakers can apply the study’s results to understand the public’s psychological state, timely adjust the relevant carbon neutrality policies, and strengthen public participation in carbon-neutral action. HSM and TAM were first used to discuss the process of forming public attention and sentiment and analyzed the internal mechanism of public attention and sentiment toward carbon neutrality. Then, based on the social media platform as the data source, statistical analysis, the Mann-Kendall method, keyword analysis, and machine learning models (BERT model, LDA model) were used to investigate the heuristic and systematic cues of posts on carbon neutrality on Sina Weibo from January 1, 2020, to August 31, 2022. The results are as follows:

1. From the results of the overall analysis, differences in attention to carbon neutrality exist due to differences in gender and geographic location of Weibo users. Men and people from economically developed regions are more concerned about carbon neutrality. Geographically, public attention in the east of the Hu line was generally higher than in the west.
2. For the heuristic cues, information source credibility can influence public attention. The results show that information provided by highly credible governments or IGOs creates high public interest in carbon neutrality and great dynamic changes in public attention toward carbon neutrality. In addition, those in the energy finance market pay great attention to carbon neutrality.
3. For the systematic cues, topics of the information content can influence public sentiment. The results show that the vast majority of the Chinese public maintains positive sentiments toward carbon neutrality, and the topics involved in carbon neutrality have different effects on public sentiment. Carbon-neutral topics related to promoting the development of green energy will result in positive feelings among the public. Carbon neutrality topics related to inadequate energy supply may evoke negative public sentiments.

Based on these research findings, we make several suggestions to policymakers to improve public attention and sentiment toward carbon neutrality and to strengthen public support for carbon neutrality. First, government departments should increase publicity on carbon-neutral policies to mitigate the cognitive lag of women and people from economically undeveloped regions. Policymakers should also consider the acceptance of women, people from economically undeveloped regions, and the movement of energy financial markets to formulate carbon-neutral policies. Second, in order to promote carbon neutrality effectively, it is important for relevant departments to prioritize the sharing of credible information on carbon neutrality from IGOs and governments, as the public increasingly values highly credible sources of information. Third, carbon-neutral policies related to promoting the development of green energy should be introduced, which can be achieved by formulating national strategies, innovating low-carbon technologies, subsidizing financial markets, and encouraging the transformation of the energy industry. Fourth, carbon-neutral policies resolving the contradiction between energy supply and demand, including smoothing energy prices and reducing policy news about energy undersupply, should be formulated.

This study also has some limitations. It is based on Weibo users’ data; however, the age distribution of Weibo users is not obvious, and the education level of the users is undisclosed or not easy to determine. Therefore, this study could not conduct further analyses on information recipients’ characteristics and the perceptions of older adults. In addition, it only considered the Chinese social media platform Sina Weibo without considering foreign social media platforms such as Twitter. In future studies, it will be helpful to compare and analyze public cognition on domestic and foreign social media platforms.

## Data availability statement

The raw data supporting the conclusions of this article will be made available by the authors, without undue reservation.

## Author contributions

BW: supervision, funding acquisition, project administration, and writing – review and editing. ZJ: conceptualization, data processing, methodology, formal analysis, writing – original draft, and writing – review and editing. DC: conceptualization, methodology, formal analysis, and writing – original draft. ZW: methodology and writing – review and editing. All authors contributed to the article and approved the submitted version.

## Funding

This research was supported by the National Social Science Fund of China (Grant No. 21BJY158).

## Conflict of interest

The authors declare that the research was conducted in the absence of any commercial or financial relationships that could be construed as a potential conflict of interest.

## Publisher’s note

All claims expressed in this article are solely those of the authors and do not necessarily represent those of their affiliated organizations, or those of the publisher, the editors and the reviewers. Any product that may be evaluated in this article, or claim that may be made by its manufacturer, is not guaranteed or endorsed by the publisher.

## References

[ref1] Abdar M. Basiri M. E. Yin J. Habibnezhad M. Chi G. Nemati S.. (2020). Energy choices in Alaska: mining people’s perception and attitudes from geotagged tweets. Renew. Sust. Energ. Rev. 124:109781. doi: 10.1016/j.rser.2020.109781 PMC10681364 38013678

[ref2] Adut A. (2012). A theory of the public sphere. Socio. Theor. 30, 238–262. doi: 10.1177/0735275112467012

[ref3] Carriquiry M. Dumortier J. Elobeid A. (2022). Trade scenarios compensating for halted wheat and maize exports from Russia and Ukraine increase carbon emissions without easing food insecurity. Nat. Food. 3, 847–850. doi: 10.1038/s43016-022-00600-0, PMID: 37117877

[ref4] Chaiken S. (1980). Heuristic versus systematic information processing and the use of source versus message cues in persuasion. J. Pers. Soc. Psychol. 39, 752–766. doi: 10.1037/0022-3514.39.5.752

[ref5] Chaiken S. Maheswaran D. (1994). Heuristic processing can bias systematic processing: effects of source credibility, argument ambiguity, and task importance on attitude judgment. J. Pers. Soc. Psychol. 66, 460–473. doi: 10.1037/0022-3514.66.3.460, PMID: 8169760

[ref6] Chaiken S. Trope Y. (1999). Dual-process theories in social psychology. Guilford, New York.

[ref7] Chen M. Gong Y. Li Y. Lu D. Zhang H. (2016). Population distribution and urbanization on both sides of the Hu Huanyong line: answering the Premier’s question. J. Geogr. Sci. 26, 1593–1610. doi: 10.1007/s11442-016-1346-4

[ref8] Chen C. F. Xu X. Arpan L. (2017). Between the technology acceptance model and sustainable energy technology acceptance model: investigating smart meter acceptance in the United States. Energy Res. Social Sci. 25, 93–104. doi: 10.1016/j.erss.2016.12.011

[ref9] Cohen B. Cowie A. Babiker M. Leip A. Smith P. (2021). Co-benefits and trade-offs of climate change mitigation actions and the sustainable development goals. Sustain. Prod. Consum. 26, 805–813. doi: 10.1016/j.spc.2020.12.034

[ref10] Davis F. D. (1989). Perceived usefulness, perceived ease of use, and user acceptance of information technology. MIS Q. 13, 319–340. doi: 10.2307/249008

[ref11] Devlin J. Chang M. W. Lee K. Toutanova K. (2018). Bert: pre-training of deep bidirectional transformers for language understanding. ArXiv:1810.04805. doi: 10.48550/arXiv.1810.04805

[ref12] Eagly A. H. Chaiken S. (1993). The psychology of attitudes. Harcourt Brace & Company, Orlando, FL

[ref13] Fergen J. Jacquet J. B. (2016). Beauty in motion: expectations, attitudes, and values of wind energy development in the rural US. Energy Res. Social Sci. 11, 133–141. doi: 10.1016/j.erss.2015.09.003

[ref14] Finance Net Official. (2022). Germany cancels 2035 carbon neutrality target. Available at: http://s.weibo.com/weibo?q=%23%E5%BE%B7%E5%9B%BD%E5%8F%96%E6%B6%882035%E5%B9%B4%E7%A2%B3%E4%B8%AD%E5%92%8C%E7%9B%AE%E6%A0%87%23 (Accessed April 12, 2023)

[ref15] Financial News Official. (2021). Why are multiple regions implementing "power rationing" measures? Can the follow-up power supply be guaranteed? How to use electricity scientifically? Available at: https://mp.weixin.qq.com/s/1H6QQO-5SLxoLTn9-_75Wg (Accessed April 12, 2023).

[ref16] Gong P. Wang L. Wei Y. Yu Y. (2022). Public attention, perception, and attitude towards nuclear power in China: a large-scale empirical analysis based on social media. J. Clean. Prod. 373:133919. doi: 10.1016/j.jclepro.2022.133919

[ref17] Ho S. S. Looi J. Chuah A. S. Leong A. D. Pang N. (2018). “I can live with nuclear energy if …”: exploring public perceptions of nuclear energy in Singapore. Energy Policy 120, 436–447. doi: 10.1016/j.enpol.2018.05.060

[ref18] Ho S. S. Yu P. Tandoc E. C. Jr. Chuah A. S. (2022). Mapping risk and benefit perceptions of energy sources: comparing public and expert mental models in Indonesia, Malaysia, and Singapore. Energy Res. Social Sci. 88:102500. doi: 10.1016/j.erss.2022.102500

[ref19] Hong D. Y. Xiao C. Y. (2007). Sociological analysis on gender difference of environmental concern. Socio. Stud. 2, 111–135. doi: 10.19934/j.cnki.shxyj.2007.02.005

[ref20] Huang H. Long R. Y. Chen H. Sun K. Li Q. W. (2022). Exploring public attention about green consumption on Sina Weibo: using text mining and deep learning. Sustain. Prod. Consum. 30, 674–685. doi: 10.1016/j.spc.2021.12.017

[ref21] Huang W. Q. Wang Q. F. Li H. Fan H. B. Qian Y. Klemes J. J. (2022). Review of recent progress of emission trading policy in China. J. Clean. Prod. 349:131480. doi: 10.1016/j.jclepro.2022.131480

[ref22] Huijts N. M. Molin E. J. Steg L. (2012). Psychological factors influencing sustainable energy technology acceptance: a review-based comprehensive framework. Renew. Sust. Energ. Rev. 16, 525–531. doi: 10.1016/j.rser.2011.08.018

[ref23] Ide T. Frohlich C. Donges J. F. (2020). The economic, political, and social implications of environmental crises. Bull. Am. Meteorol. Soc. 101, E364–E367. doi: 10.1175/bams-d-19-0257.1

[ref24] International Business Daily Official. (2021). The 1st China digital carbon neutrality summit. Available at: (https://m.weibo.cn/1649173367/4678715235371499).

[ref25] James O. Petersen C. (2018). International rankings of government performance and source credibility for citizens: experiments about e-government rankings in the UK and the Netherlands. Public Manag. Rev. 20, 469–484. doi: 10.1080/14719037.2017.1296965

[ref26] Lawrence M. G. Schafer S. Muri H. Scott V. Oschlies A. Vaughan N. E.. (2018). Evaluating climate geoengineering proposals in the context of the Paris agreement temperature goals. Nat. Commun. 9, 3734–3719. doi: 10.1038/s41467-018-05938-3, PMID: 30213930 PMC6137062

[ref27] Lee H. Chung N. (2019). Assessing the factors that drive consumers’ intention to continue using online travel agencies: a heuristic-systematic model perspective. Asia Pac. J. Inf. Syst. 29, 468–488. doi: 10.14329/apjis.2019.29.3.468

[ref28] Li X. Hu Z. G. Cao J. H. Xu X. (2022). The impact of environmental accountability on air pollution: a public attention perspective. Energy Policy 161:112733. doi: 10.1016/j.enpol.2021.112733

[ref29] Li L. Liu X. Zhang X. (2021). Public attention and sentiment of recycled water: evidence from social media text mining in China. J. Clean. Prod. 303:126814. doi: 10.1016/j.jclepro.2021.126814

[ref30] Li P. Sun W. Zhang Z. He Y. Wang Y. (2022). Forecast of renewable energy penetration potential in the goal of carbon peaking and carbon neutrality in China. Sustain. Prod. Consum. 34, 541–551. doi: 10.1016/j.spc.2022.10.007

[ref31] Li J. Wang J. Zhang J. Zhang J. Kong H. (2021). Dynamic changes of vegetation coverage in China-Myanmar economic corridor over the past 20 years. Int. J. Appl. Earth Obs. Geoinf. 102:102378. doi: 10.1016/j.jag.2021.102378

[ref32] Linzenich A. Zaunbrecher B. S. Ziefle M. (2020). “Risky transitions?” risk perceptions, public concerns, and energy infrastructure in Germany. Energy Res. Social Sci. 68:101554. doi: 10.1016/j.erss.2020.101554

[ref33] Liu X. Hu W. (2019). Attention and sentiment of Chinese public toward green buildings based on Sina Weibo. Sust. Cities Soc. 44, 550–558. doi: 10.1016/j.scs.2018.10.047

[ref34] May A. M. McGarvey M. G. Gustafson C. R. Mieno T. (2021). Gender, environmental issues and policy: an examination of the views of male and female economists. Ecol. Econ. 182:106877. doi: 10.1016/j.ecolecon.2020.106877

[ref35] Park E. (2019). Positive or negative? Public perceptions of nuclear energy in South Korea: evidence from big data. Nucl. Eng. Technol. 51, 626–630. doi: 10.1016/j.net.2018.10.025

[ref36] Piselli C. Colladon A. F. Segneri L. Pisello A. L. (2022). Evaluating and improving social awareness of energy communities through semantic network analysis of online news. Renew. Sust. Energ. Rev. 167:112792. doi: 10.1016/j.rser.2022.112792

[ref37] Sarkar J. G. Sarkar A. Sreejesh S. (2023). Developing responsible consumption behaviours through social media platforms: sustainable brand practices as message cues. Inf. Technol. People 36, 532–563. doi: 10.1108/ITP-01-2021-0044

[ref38] Schmidt-Traub G. Kroll C. Teksoz K. Durand-Delacre D. Sachs J. D. (2017). National baselines for the sustainable development goals assessed in the SDG index and dashboards. Nat. Geosci. 10, 547–555. doi: 10.1038/ngeo2985

[ref39] Shi Y. Y. Wei Z. X. Shahbaz M. (2023). Analyzing the co-evolutionary dynamics of consumers’ attitudes and green energy technologies based on a triple-helix model. Renew. Sust. Energ. Rev. 171:113009. doi: 10.1016/j.rser.2022.113009

[ref40] Shrivastava A. Jain G. Kamble S. S. Belhadi A. (2021). Sustainability through online renting clothing: circular fashion fueled by instagram micro-celebrities. J. Clean. Prod. 278:123772. doi: 10.1016/j.jclepro.2020.123772

[ref41] Sigmond M. Fyfe J. C. Swart N. C. (2018). Ice-free arctic projections under the Paris agreement. Nat. Clim. Chang. 8, 404–408. doi: 10.1038/s41558-018-0124-y

[ref42] Son J. Lee J. Oh O. Lee H. K. Woo J. (2020). Using a heuristic-systematic model to assess the twitter user profile’s impact on disaster tweet credibility. Int. J. Inf. Manag. 54:102176. doi: 10.1016/j.ijinfomgt.2020.102176

[ref43] Trumbo C. W. (2002). Information processing and risk perception: an adaptation of the heuristic-systematic model. J. Commun. 52, 367–382. doi: 10.1111/j.1460-2466.2002.tb02550.x

[ref44] Weibo Official. (2021). 2020 Weibo user development report. Available at: https://weibo.com/1642909335/K5OFGDin3 (Accessed 6 January 2023).

[ref45] Wu Z. Huang X. Chen R. Mao X. Qi X. (2022). The United States and China on the paths and policies to carbon neutrality. J. Environ. Manag. 320:115785. doi: 10.1016/j.jenvman.2022.115785, PMID: 36056478

[ref46] Xiao C. Hong D. (2010). Gender differences in environmental behaviors in China. Popul. Env. 32, 88–104. doi: 10.1007/s11111-010-0115-z

[ref47] Xu T. Kang C. Zhang H. (2022). China's efforts towards carbon neutrality: does energy-saving and emission-reduction policy mitigate carbon emissions? J. Environ. Manag. 316:115286. doi: 10.1016/j.jenvman.2022.115286, PMID: 35658256

[ref48] Xu Z. C. Li Y. J. Chau S. N. Dietz T. Li C. B. Wan L. W.. (2020). Impacts of international trade on global sustainable development. Nat. Sustain. 3, 964–971. doi: 10.1038/s41893-020-0572-z

[ref49] Yang P. J. Peng S. Benani N. Dong L. Y. Li X. M. Liu R. P.. (2022). An integrated evaluation on China's provincial carbon peak and carbon neutrality. J. Clean. Prod. 377:134497. doi: 10.1016/j.jclepro.2022.134497

[ref50] Zhang W. Watts S. A. (2008). Capitalizing on content: information adoption in two online communities. J. Assoc. Inf. Syst. 9, 73–94. doi: 10.17705/1jais.00149

[ref51] Zhang K. Z. Zhao S. J. Cheung C. M. Lee M. K. (2014). Examining the influence of online reviews on consumers' decision-making: a heuristic–systematic model. Decis. Support. Syst. 67, 78–89. doi: 10.1016/j.dss.2014.08.005

[ref52] Zheng S. Wang J. Sun C. Zhang X. Kahn M. E. (2019). Air pollution lowers Chinese urbanites’ expressed happiness on social media. Nat. Hum. Behav. 3, 237–243. doi: 10.1038/s41562-018-0521-2, PMID: 30953012

